# Development of a Scrub Typhus Diagnostic Platform Incorporating Cell-Surface Display Technology

**DOI:** 10.3389/fimmu.2021.761136

**Published:** 2021-10-11

**Authors:** Chih-Chi Liao, Chih-Hsuan Tsai, Huei-Ru Lo, Pey-Ru Lin, Chang-Chi Lin, Yu-Chan Chao

**Affiliations:** ^1^ Institute of Molecular Biology, Academia Sinica, Taipei, Taiwan; ^2^ Institute of Preventive Medicine, National Defense Medical Center, Taipei, Taiwan; ^3^ Department of Entomology, National Chung Hsing University, Taichung, Taiwan; ^4^ Department of Entomology, College of Bioresources and Agriculture, National Taiwan University, Taipei, Taiwan; ^5^ Department of Plant Pathology and Microbiology, College of Bioresources and Agriculture, National Taiwan University, Taipei, Taiwan

**Keywords:** baculovirus surface display, cell-based ELISA, *Orientia tsutsugamushi*, scrub typhus, serological diagnosis

## Abstract

Scrub typhus (ST), also known as tsutsugamushi disease and caused by rickettsia *Orientia tsutsugamushi*, is an underestimated fatal epidemic in the Asia-Pacific region, resulting in a million human infections each year. ST is easily misdiagnosed as clinical diagnosis is based on non-specific skin eschar and flu-like symptoms. Thus, the lack of accurate, convenient, and low-cost detection methods for ST poses a global health threat. To address this problem, we adopted baculovirus surface-display technology to express three variants of TSA56, the major membrane antigen of *O. tsutsugamushi*, as well as the passenger domain of ScaC (ScaC-PD), on insect Sf21 cell surfaces rather than biosafety level 3 bacteria in an enzyme-linked immunosorbent assay (ELISA). Recombinant TSA56 and ScaC-PD were all properly expressed and displayed on Sf21 cells. Our cell-based ELISA comprising the four antigen-displaying cell types interacted with monoclonal antibodies as well as serum samples from ST-positive field-caught rats. This cell-based ELISA presented high accuracy (96.3%), sensitivity (98.6%), and specificity (84.6%) when tested against the ST-positive rat sera. Results of a pilot study using human sera were also highly consistent with the results of immunofluorescence analyses. By adopting this approach, we circumvented complex purification and refolding processes required to generate recombinant *O. tsutsugamushi* antigens and reduced the need for expensive equipment and extensively trained operators. Thus, our system has the potential to become a widely used serological platform for diagnosing ST.

## Introduction

Scrub typhus (ST), also known as tsutsugamushi disease, was endemic in the Asia-Pacific region (the so-called “tsutsugamushi triangle”), but it has now spread worldwide due to globalization, with one million cases each year (1, 2). The causative pathogen is *Orientia tsutsugamushi*, a gram-negative and obligate intracellular rickettsial bacterium that is transmitted to warm-blooded animals by bites of *Leptotrombidium* mite larvae (chiggers) (1). Initial ST symptoms include headache, myalgia, fever, rash, vomiting, and abdominal pain. Without accurate and timely antibiotic treatment—such as with doxycycline, tetracycline, chloramphenicol, rifampicin, or azithromycin—fatality can reach 30% (3). However, clinical diagnosis of ST is problematic because of the broad-spectrum flu-like symptoms, relying instead on observation of skin eschar at the bite site. Even so, ST can still be easily misdiagnosed as dengue fever, typhus, or malaria (4). Thus, a rapid and accurate diagnostic method is vital to tackle the ST epidemic.

Confirmatory ST diagnosis can be achieved by molecular or serological methods. Molecular diagnosis is based on polymerase chain reaction (PCR) of *O. tsutsugamushi* genes such as *p56* (TSA56), *p47*, or *groEL* (5). Such diagnoses necessitate initial isolation or culture of *O. tsutsugamushi*, so can only be applied during the acute infection phase (5, 6). Serological diagnoses encompass the Weil-Felix test, immunofluorescence assay (IFA), and enzyme-linked immunosorbent assay (ELISA) to detect antibodies against *O. tsutsugamushi* in a patient’s serum. Of these three latter tests, the Weil-Felix test is the cheapest but it lacks specificity and sensitivity. IFA, generally regarded as the gold standard diagnostic approach, requires experienced personnel and expensive equipment (3, 7). ELISA is more convenient and can be conducted at moderate costs (3, 8). The primary drawback of both molecular and serological methods is that experiments using live *O. tsutsugamushi* must be performed in biosafety level 3 (BSL-3) laboratories, greatly limiting accessibility to diagnostic approaches, especially in developing countries where ST is the greatest threat (7, 9).

To circumvent using live bacteria in ELISA, *Escherichia coli* can be employed to express *O. tsutsugamushi* TSA56 protein, which can then be utilized for ELISA analysis upon purification (9–14). TSA56 antigen is the most abundant protein on *O. tsutsugamushi* cell surfaces (accounting for 15% total protein), and it is responsible for attachment to host cells and for inducing phagocytosis. Consequently, TSA56 is highly immunogenic (10). However, in animal immunization experiments, a single variant of TSA56 only provided partial (11-56%) protection against challenge by various strains of *O. tsutsugamushi* (10), indicating that antibodies against TSA56 are strain-specific. Thus, recombinant TSA56 from three prototype strains (Gilliam, Karp, and Kato) are typically incorporated into diagnostic systems to prevent false-negative detections (7). To develop a more universal ELISA system involving further antigens, surface cell antigen (Sca) proteins from *O. tsutsugamushi* have also been cloned and expressed for ELISA, with ScaC—an autotransporter protein responsible for host cell adhesion, aggregation, invasion, and biofilm formation (15)—showing the highest sequence similarity among different strains, thus potentially representing an appropriate target antigen for ST detection (16). *E. coli*-based antigen cloning has resolved the biohazard issue arising from using *O. tsutsugamushi*, but the resulting recombinant proteins are mostly insoluble and so require time-consuming dialysis and refolding steps after complex purification. Moreover, an additional process of antigen coating on microtiter plates is required.

Baculoviruses are large rod-shaped DNA viruses from Family Baculoviridae (17). Autographa californica multiple nucleopolyhedrovirus (AcMNPV), the type species of alphabaculoviruses, has long been used to express proteins in insect cells because its strong late promoters can produce recombinant proteins at scale and it can accommodate extensive eukaryotic post-translational modification and protein oligomerization (18). Baculovirus can display a recombinant protein on the plasma membrane of insect cells upon virus infection once the target protein contains a transmembrane (TM) domain or is fused with the TM from GP64, the surface glycoprotein of AcMNPV (19). Insect cells displaying target protein can serve as a source of antigen for serological assay of a viral disease (20) or in blood typing (21). Since antigens on insect cells are highly accessible to serum antibodies, further purification is not necessary. Furthermore, baculoviruses and insect cells can be handled in BSL-1 laboratories (22). These features make insect cells that display recombinant antigens *via* baculovirus ideal for replacing infectious pathogens in serological assays such as ELISA.

In this study, we utilized a baculovirus-insect cell expression system to display high levels of *O. tsutsugamushi* surface antigens on recombinant virus-infected insect cells to develop a cell-based ELISA system for rapid and convenient ST diagnosis of patient sera. The system circumvents the need for complex protein purification and antigen-coating processes, and our results show that the cell-based ELISA system exhibits high accuracy and specificity for ST detection using serum collected from animals and humans.

## Materials and Methods

### Cell Lines and Cell Culture

We used *Spodoptera frugiperda* cell line IPLB-Sf21 (Sf21). The cells were cultured in TC100 insect medium (US Biological, T2000) supplemented with 10% fetal bovine serum (FBS; Gibco, 10438-026) at 26°C. Cells were passaged every 3 days.

### Construction of Transfer Vectors

The backbone vector pAB-pEG-hhp10-HM6H-6MC was constructed by replacing the *polyhedrin* promoter (*p-polh*) at multiple cloning sites in pBacPAK8 (Clontech Laboratories Inc.) with the *hr1-hsp70-p10* dual promoter and by inserting an *EGFP* reporter gene driven by the *pag* promoter (23, 24) (*p-pag*) upstream of multiple cloning sites. Within the multiple cloning sites, the coding sequence of a honeybee melittin (HM) signal peptide followed by a hexameric Histidine tag and the TM and cytoplasmic tailed domains of GP64 (6MC) were inserted downstream of the *p10* promoters. DNA fragments of all inserted components were amplified by PCR and then ligated by In-Fusion HD Cloning Kit (TaKaRa Bio Inc., 639650). DNA sequences of TSA56 target proteins (i.e., from the Gilliam, Karp, and Kato strains) were amplified by PCR from pET101 plasmids inserted with the respective TSA56 coding sequence (25). Passenger domain of ScaC (ScaC-PD) was amplified from genomic DNA of the Gilliam strain. Fragments of the backbone vector and inserts were ligated by In-Fusion HD Cloning Kit to generate transfer vectors pAB-pEG-hhp10-HM6H-Gilliam, pAB-pEG-hhp10-HM6H-Karp, pAB-pEG-hhp10-HM6H-Kato and pAB-pEG-hhp10-HM6H-ScaC-PD-6MC, respectively. Primers used in this study are presented in **Table 1**.

**Table 1:** Primers used in this study.

Primer	DNA sequence (5’ - 3’)	Reference
tsa56 Gilliam & Karp forward	CACCATCACCATCACATAGAATTGGGTGAGGAAGG	This study
tsa56 Gilliam & Karp reverse	CGGATCAATTAATTACTAGAAGTTATAGCGTACAC	This study
tsa56 Kato forward	CACCATCACCATCACATAGAATTGGGGGATGAAGG	This study
tsa56 Kato reverse	CGGATCAATTAATTACTAGAAGTTATAGCGCACAC	This study
ScaC FL/PD forward	CACCATCACCATCACGGAAAATATCCTCAGGCAG	This study
ScaC PD reverse	ATGACCAAACATGAAAATATCTCTGATATAGTTTAATTTAACG	This study
pAB-pEG-hhp10-HM forward 0518	TAATTAATTGATCCGGGTTATTAGTAC	This study
pAB-pEG-hhp10-HM reverse 0518	GTGATGGTGATGGTGATG	This study
pAB-pEG-hhp10-HM CTD+ forward	TTCATGTTTGGTCATGTAGT	This study

### Recombinant Baculovirus Construction

Individual transfer vectors were co-transfected into Sf21 cells with a modified baculovirus genomic DNA, flashBac™ ULTRA (Mirus, MIR 6140), according to the guidelines for TransIT^®^-Insect Transfection Reagent (Mirus, MIR 6100) to obtain the recombinant viruses (P0 virus). Single virus clones (P1 virus) were obtained by end-point dilution of the P0 virus. Single viruses with the best protein expression, as assessed by Western blot, were selected and amplified in Sf21 cells (P2 virus) for the following cell-based assays.

### Virus Titer Determination by 50% Tissue Culture Infective Dose (TCID_50_) Assay

Viral stock solutions were serially diluted 10^-1^ to 10^-10^-fold and added to Sf21 cells seeded on microtiter plates. The plates were subjected to centrifugation at 2000 rpm for 30 min at 26 °C. After incubation for 5 days at 26°C, plates were observed under fluorescence microscopy. TCID_50_ titers were calculated from the number of wells exhibiting green fluorescence signals according to the Reed–Muench method (26).

### Western Blotting Analysis

Sf21 cells were infected with individual recombinant viruses for 2 days. Then the cells were collected, washed once with Dulbecco’s phosphate-buffered saline (DPBS), and lysed in RIPA buffer (Thermo Scientific, 89901). The cell lysates were separated by sodium dodecyl sulfate-polyacrylamide gel electrophoresis (SDS-PAGE) in a 10% gel and then transferred to polyvinylidene fluoride membrane (PVDF; GE Healthcare Life Sciences, 10600021) with 1X Western Transfer buffer (Omics Bio, IB3352). Western blotting was performed with primary mouse anti-His antibody (1:2000; Bio-Rad, MCA1396) and secondary goat anti-mouse IgG (1:5000; Jackson ImmunoResearch Laboratories Inc., 115-035-003). Expression of GAPDH in cell lysates was determined as an internal control using primary rabbit anti-GAPDH antibody (1:10000; GeneTex, GTX100118) and secondary goat anti-rabbit IgG antibody (1:10000; Jackson ImmunoResearch Laboratories Inc., 111-035-003).

### IFA to Validate Surface Display of Recombinant Proteins

IFA was performed according to previous studies (20, 24, 27). Sf21 cells (1 × 10^4^ cells/well) were seeded into the wells of Millicell^®^ EZ chamber slides (Millipore, PEZGS0816) and infected with individual recombinant baculoviruses at multiplicity of infection (MOI)=1 for 2 days. The infected cells were washed once with DPBS and then fixed with 4% paraformaldehyde (Electron Microscopy Science, 15710) for 15 min at room temperature. After blocking with ChonBlock Blocking/Sample Dilution Buffer (Chondrex, 9068), the cells were incubated with primary mouse anti-His antibody (1:200; Bio-Rad, MCA1396) for 2 h at room temperature. After three washes with DPBS plus 0.1% Tween 20 (DPBST), the cells were incubated with Alexa Fluor 555 Goat anti-Mouse IgG (H+L) secondary antibody (1:5000; Invitrogen, A21422) and counterstained with 49,69-diamidino-2-phenylindole (DAPI) (Thermo Fisher Scientific, D1306). Distribution of fluorescence signal was observed under Zeiss laser confocal microscopy (LSM780).

### Rat Serum Samples

A total of 69 serum samples were collected from field-caught rats on the Penghu and Lanyu islands of Taiwan. The 28 sera from Penghu were obtained from *Rattus norvegicus* or *Rattus losea* from 11-15 March 2019 and have been designated PH20-2, 3, 4, 5, 6, 7, 20, 21, 22, 23, 32, 39, 40, 41, 44, 45, 46, 48, 49, 50, 51, 52, 53, 54, 55, 56, 57, and 62, respectively. All 41 sera from Lanyu were collected on 17 April 2019 from *Rattus mindanaoensis* and have been designated OI07-2, 3, 4, 5, 6, 7, 8, 9, 10, 11, 12, 13, 14, 15, 16, 17, 18, 19, 20, 21, 22, 23, 24, 25, 26, 27, 28, 29, 30, 31, 32, 33, 34, 35, 36, 37, 38, 39, 40, 41, and 42, respectively. All sera were confirmed as displaying ST antibody reaction by standard IFA. For the negative control sera, a total of 13 sera were used, including 4 normal rat sera collected from Narl : LE (Long Evans) rats that were purchased from the Taiwan National Laboratory Animal Center (designated NRS-1, 2, 3, and 4) and 9 IFA-confirmed negative sera collected from field-caught rats in Tainan and Taichung of Taiwan (designated TN08-35, 38, 39, 41, 44, 45, 47, 48, and TC01-04). Animal experiments were approved by the Institutional Animal Care and Use Committee (IACUC) of the Institute of Preventive Medicine, National Defense Medical Center, Taiwan, with approval number AN-108-03.

### Human Serum Samples

Nine human serum samples were collected by the Tri-Service General Hospital and have been designated as 18A001, 18A002, 18A003, 18A004, 18A005, 18A006, 19A002, 19A004, and 19A008. These sera were isolated from suspected ST patients. Samples 18A001, 18A002, 18A004, 18A006, and 19A002 tested negative for *O. tsutsugamushi* antibodies by IFA using L929 cells infected with *O. tsutsugamushi* (see below), whereas samples 18A003, 18A005, 19A004, and 19A008 tested positive. The study protocol was reviewed and approved by the Institutional Review Board of Tri-Service General Hospital (No. 1-106-05-158) and written informed consent was obtained from all the patients.

### IFA of *O. tsutsugamushi* Antibodies in Rat Serum Samples

We adopted a method described previously (14, 28). In brief, mouse L929 cells were infected individually with *O. tsutsugamushi* strains Gilliam, Karp, or Kato. Cells infected by each strain were mixed and spread on glass slides before fixing with acetone at -20°C for 15 min. Rat serum samples were diluted at 1:40 in PBS and applied to the slides. After incubation in a humidified chamber at 37°C for 30 min, the slides were washed three times with PBS (5 min/each time), before adding secondary antibody Goat-anti-rat IgG-Flour 488 (Leadgene Biomedical Inc., 24701) diluted 1:100. Slides were incubated again in a humid chamber at 37°C for 30 min, washed with PBS, and fitted with coverslips. Positive *O. tsutsugamushi* antibody reactions were verified based on green fluorescence emission signal under fluorescence microscopy (Olympus IX71, Tokyo, Japan) and graded empirically by viewers based on the fluorescence intensity.

### Cell-Based ELISA

Our cell-based ELISA approach has been described previously (20, 24). In brief, Sf21 cells (4 × 10^4^ cells/well) seeded in a 96-well microtiter plate were infected with recombinant baculoviruses using an MOI=1. At 2 days post infection (d.p.i.), plated cells were washed with DPBS and fixed with 4% paraformaldehyde (Electron Microscopy Science, 15710). The plates were blocked by incubating with ChonBlock Blocking/Sample Dilution Buffer (Chondrex, 9068) for 1 h at room temperature. Antibodies or serum samples were diluted (1:100) in blocking buffer and added to the plates before incubation for 2 h at room temperature. Antibodies used herein included anti-His antibody (Bio-Rad, MCA1396) and anti-TSA56 monoclonal antibodies (Yao-Hong Biotechnology, YH560001, YH560010, and YH560012). After washing three times with PBST, 50 μl horseradish peroxidase (HRP)-conjugated goat anti-mouse IgG antibody (Jackson ImmunoResearch Laboratories Inc., 115-035-003), goat anti-rat IgG antibody (Jackson ImmunoResearch Laboratories Inc., 112-035-003), or goat anti-human IgG antibody (Jackson ImmunoResearch Laboratories Inc., 109-035-097) at a dilution of 1:5,000 in 3% bovine serum albumin (BSA) in PBST or ChonBlock Detection Antibody Dilution Buffer (Chondrex, 90681) was added to each well and incubated for 1 h at room temperature. Plated cells were then washed three times with PBST, before adding 3, 3’, 5, 5’-tetramethylbenzidine (TMB) substrate (Surmodics, TMBS-0100-01) for 10 min. Coloring reactions were stopped by adding 2M sulfuric acid, and ELISA absorbance was measured at 450 nm. The cutoff for ELISA readout was determined as the mean plus two standard deviations of the absorbance of negative sera according to previous references (10, 16, 29).

To determine the specificity of cell-based ELISA, each of six rat sera that tested positive to the four recombinant antigens were two-fold serially diluted from 1:100 to 1:800 and applied to cell-based ELISA with the corresponding antigen. Another six ST-positive sera were randomly selected from sera that tested positive to any one of the recombinant antigens and they were applied to cell-based ELISA using Sf21 cells infected with wild-type baculovirus as antigens. For each test, five ST-negative rat sera were included as negative controls. Antibody titer against an individual antigen was determined for each positive serum sample as the reciprocal of the serum dilution giving twice the signal of the mean of negative controls (30).

### Statistical Analysis

At least three replicates were performed for all cell-based ELISA experiments. ELISA reads are shown as mean ± standard deviation (error bars). For cell-based ELISA using anti-His and anti-TSA56 antibodies, two-tailed *t*-tests were performed in Excel 2019 (Microsoft). For cell-based ELISA on serum samples, two-tailed Welch’s *t*-tests, linear regression analysis, and correlation analysis were performed on GraphPad Prism 9 (GraphPad Software Inc.).

## Results

### Construction of Transfer Vectors to Create Recombinant Baculoviruses Expressing *O. tsutsugamushi* Antigens

To create recombinant baculoviruses expressing *O. tsutsugamushi* antigens, we constructed transfer vectors hosting sequences encoding TSA56 and ScaC-PD. The structures of these target proteins were analyzed according to respective references in the Uniprot online protein database (https://www.uniprot.org/) to design appropriate cloning strategies.

**Figure 1:**
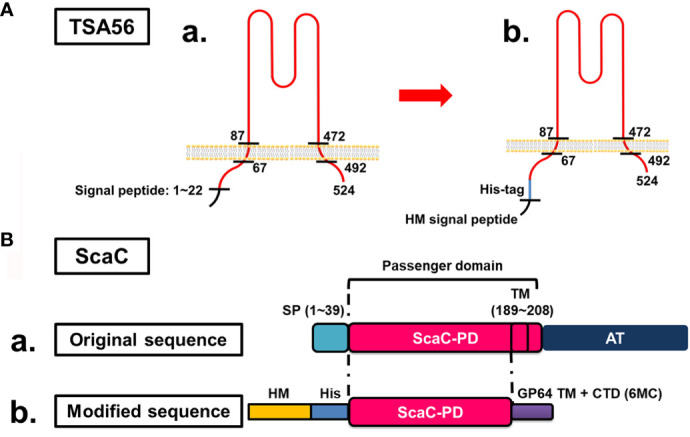
Cloning strategies for surface display of recombinant TSA56 and ScaC-PD using a baculovirus-insect cell system. **(A)** Structure and strategy for displaying TSA56 protein on Sf21 cells. **(A-a)** TSA56 protein has a signal peptide from residues 1 to 22 and two TM domains (residues 67 to 87 and 472 to 492, respectively). **(A-b)** The signal peptide was replaced by an HM signal peptide and a His-tag, but the rest of the protein was preserved. **(B)** Structure and strategy for displaying ScaC-PD on Sf21 cells. **(B-a)** ScaC contains a signal peptide (SP), a passenger domain (PD), and an autotransporter (AT) domain. **(B-b)** The signal peptide (SP) was replaced by an HM signal peptide (HM) and a His-tag (His), and the TM-truncated passenger domain was fused with the TM and cytoplasmic-tailed domain (CTD) of GP64 protein (6MC) from baculovirus.

TSA56 is a monomer possessing two TM domains (from amino acid residues 67 to 87 and 472 to 592, respectively) **(** **Figure 1A–a** **)**. To retain the natural conformation of TSA56 which may have the potential for ST vaccine application (20, 27), we preserved full-length TSA56 apart from its signal peptide. The signal peptide was replaced by an HM signal peptide that can translocate the recombinant protein to the plasma membrane of insect Sf21 cells **(** **Figure 1A–b** **)**. Although TSA56 is widely used as an antigen for ST diagnosis, strain-specific characteristics may impair detection sensitivity. Consequently, we expressed TSA56 from the Gilliam, Karp, and Kato strains. Furthermore, we also targeted ScaC as antigen, since it may be more universally recognized by antibodies elicited by *O. tsutsugamushi* strains, thereby limiting the potential for strain-specific complications due to TSA56.

ScaC comprises a signal peptide, a passenger domain, and a autotransporter domain, with this latter facilitating folding of the passenger domain **(** **Figure 1B-a** **)**. In this study, we used ScaC from the Gilliam strain. TMPRED (https://embnet.vital-it.ch/software/TMPRED_form.html) predicted a TM domain (residues 189 to 208) within the passenger domain of ScaC. We adopted a strategy employed previously for cell-based ELISA detection of Porcine epidemic diarrhea virus (PEDV) (20) and fused the ScaC passenger domain (excluding the TM) with the TM and cytoplasmic-tailed domain (CTD) of GP64 protein (6MC) to make the protein protrude from the plasma membrane of Sf21 cells (**Figure 1B-b**).

**Figure 2:**
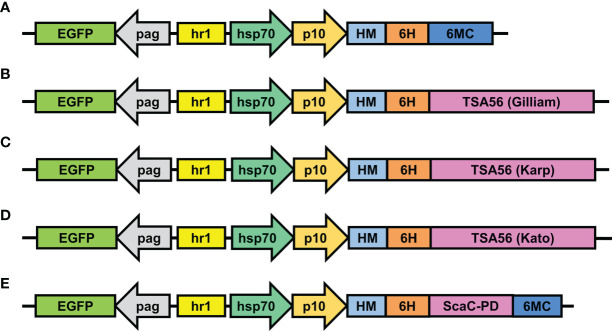
Schematic illustrations of transfer vectors used to construct recombinant baculoviruses. **(A)** The empty vector pAB-pEG-hhp10-HM-6H-6MC prior to *TSA56* or *ScaC*-PD gene insertion. *TSA56* from the Gilliam, Karp, or Kato strains, or *ScaC*-PD from Gilliam, were inserted into the vector, generating pAB-pEG-hhp10-HM6H-Gilliam **(B)**, pAB-pEG-hhp10-HM6H-Karp **(C)**, pAB-pEG-hhp10-HM6H-Kato **(D)**, and pAB-pEG-hhp10-HM6H-ScaC-PD-6MC **(E)**, respectively. EGFP, EGFP reporter gene; pag, *pag* promoter; hr1, homologous region; hsp70, *heat shock protein 70* promoter; p10, *p10* promoter; HM, honeybee melittin signal peptide; 6H, hexameric histidine tag; 6MC, transmembrane domain and cytoplasmic-tailed domain of baculovirus GP64 protein.

Expression constructs were inserted in pAB-pEG-hhp10-HM6H-6MC vector, which consists of an *EGFP* reporter gene driven by the *pag* promoter and a composite *hr1*-*hsp70*-*p10* promoter composed of a *heat shock protein 70* promoter (enhanced by the *hr1* sequence) and the baculovirus late promoter *p10* to drive foreign gene expression **(** **Figure 2A** **)**. Recombinant TSA56 and ScaC-PD, both conjugated sequentially with the HM signal peptide and a His-tag at the N-terminal, were placed downstream of the *p10* promoter. For the three TSA56 variants (from the Gilliam, Karp, and Kato strains), the 6MC vector element was removed, allowing the recombinant proteins to be anchored *via* their own two TM domains **(** **Figures 2B–D** **)**. Recombinant ScaC-PD was further fused with the 6MC vector element to achieve membrane anchorage **(** **Figure 2E** **)**.

### Confirming Expression and Cell-Surface Display of Recombinant Antigens

**Figure 3:**
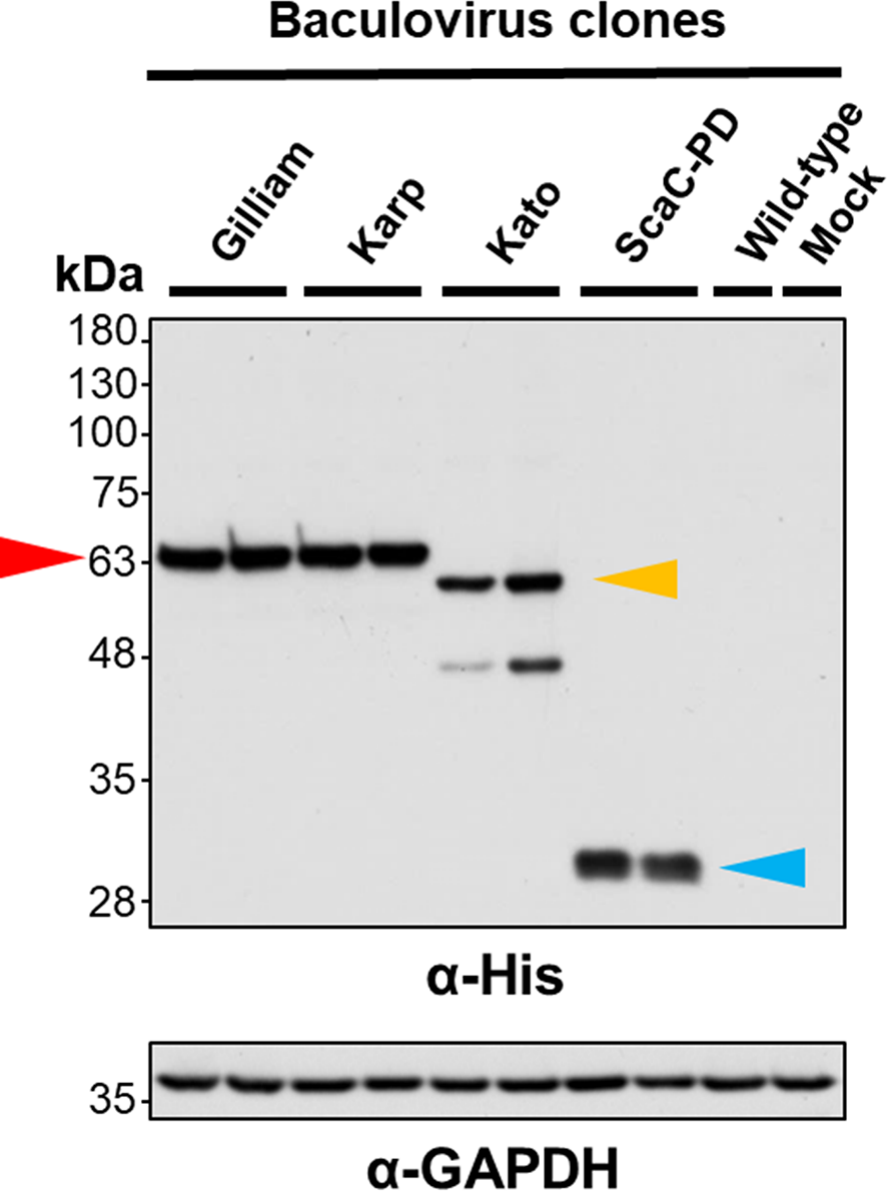
Validation of recombinant protein expression by Western blot analysis. Sf21 cells were infected with recombinant baculoviruses expressing TSA56 or ScaC-PD for 2 days. Expression of target proteins was assessed by Western blotting using anti-His antibody. GAPDH expression was determined as a loading control using anti-GAPDH antibody. Red arrow: TSA56 protein of the Gilliam and Karp strains. Yellow arrow: TSA56 protein of the Kato strain. Blue arrow: ScaC-PD. Wild-type: cells infected with wild-type baculovirus lacking foreign gene expression. Mock: cells not subjected to virus infection.

Following construction of each of the recombinant viruses, we isolated viral clones showing the highest protein expression for further cell-based assay. To validate expression and cell-surface display of recombinant proteins, we infected Sf21 cells with selected viruses and wild-type baculovirus at an MOI of 1 and harvested the infected cells at 2 d.p.i. for Western blot analysis and IFA. Western blot with anti-His antibody revealed that TSA56 from the Gilliam and Karp strains had a molecular weight of ~63 kDa, indicating post-translational modification of the recombinant proteins. We observed two bands upon Western blotting of recombinant TSA56 from the Kato strain, a major one of ~60 kDa and a minor one of <48 kDa. ScaC-PD exhibited a molecular weight of ~30 kDa **(** **Figure 3** **)**.

**Figure 4:**
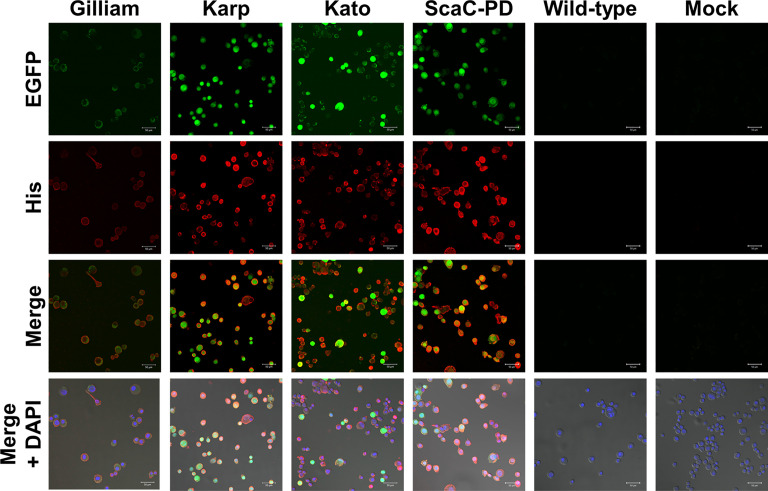
Immunofluorescence assay to confirm surface display of target proteins by Sf21 cells. Sf21 cells infected with recombinant baculoviruses carrying TSA56 or ScaC-PD gene constructs emitted green fluorescence arising from co-expression of the *EGFP* reporter gene. Cell surface display of recombinant TSA56 or ScaC-PD by the Sf21 cells was examined by immunostaining using primary anti-His antibody and secondary Alexa Fluor 555 antibody (red fluorescence). DAPI: nuclear counterstain (blue fluorescence). Wild-type: cells infected with wild-type baculovirus. Mock: cells not subjected to virus infection. Scale bar: 50 μm.

Infected cells displayed green fluorescence under IFA due to expression of EGFP driven by the *pag* promoter **(** **Figure 2** **)**, and fluorescence intensity varied according to infection stage. We further immunostained the cells with anti-His antibody followed by Alexa Fluor 555 dye to confirm display of the target proteins on the insect cells. As revealed by confocal microscopy, anti-His antibody signal (red fluorescence) surrounded the infected Sf21 cells. In contrast, Sf21 cells infected with wild-type baculovirus or not subjected to virus infection showed no red fluorescence signal **(** **Figure 4** **)**. These results confirmed that the target proteins were expressed by cells infected with recombinant baculoviruses and those proteins were primarily displayed on cell surfaces.

### Establishment of an Insect Cell-Based ELISA That Efficiently Express *O. tsutsugamushi* Antigens

**Figure 5:**
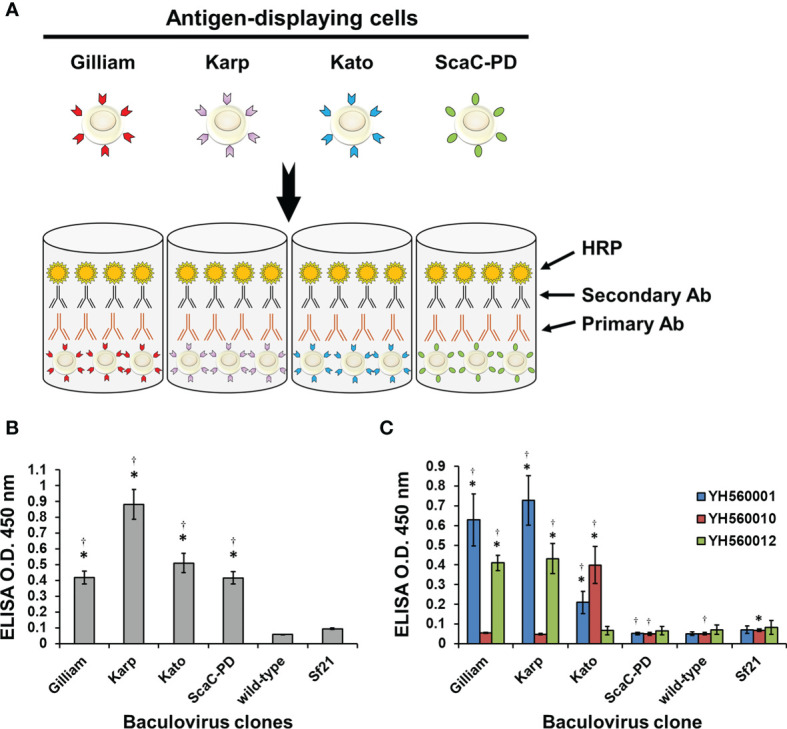
Validation of our cell-based ELISA using anti-His and anti-TSA56 monoclonal antibodies. **(A)** Schematic illustration of our cell-based ELISA. Sf21 cells infected by baculoviruses expressing TSA56 or ScaC-PD gene constructs were seeded into micro-well plates and treated with primary antibodies (Primary Ab) before adding secondary antibodies (Secondary Ab) that are conjugated with horseradish peroxidase (HRP). Cell-based ELISA treated with anti-His antibody **(B)** or commercial anti-TSA56 monoclonal antibodies YH560001, YH560010, or YH560012 **(C)**. Wild-type: cells infected with wild-type baculovirus. Sf21: Sf21 cells not subjected to virus infection. ELISA reads are expressed as mean ± standard deviation (SD) of three replicates. Statistical significance was determined by Student’s *t*-test. ^*^significant difference (*p*<0.05) *versus* the value for wild-type. ^†^significant difference (*p* < 0.05) *versus* non-infected Sf21 cells.

After confirming expression and cell surface display of *O. tsutsugamushi* antigens by the recombinant baculoviruses, we used the infected Sf21 cells to establish our cell-based ELISA system. Sf21 cells infected by baculoviruses expressing one of the three different TSA56 antigens or the ScaC-PD antigen were seeded into wells of micro-well plates for ELISA detection **(** **Figure 5A** **)**. Initially, we used anti-His antibody and three commercial anti-TSA56 monoclonal antibodies to validate that the recombinant proteins displayed on the cells could be recognized by them. All of the recombinant baculovirus-infected Sf21 cells exhibited significant ELISA signals upon reacting with anti-His antibody relative to cells infected by wild-type baculovirus or cells lacking virus infection **(** **Figure 5B** **)**. We used anti-TSA56 monoclonal antibodies (YH560001, YH560010, and YH560012) to confirm antigen recognition. YH560001 significantly recognized all three TSA56 variants, whereas YH560010 significantly recognized TSA56 from the Kato strain and YH560012 specifically recognized TSA56 from the Gilliam and Karp strains **(** **Figure 5C** **)**. The results indicate that these anti-TSA56 antibodies recognize different epitopes on the protein and that the recombinant proteins we displayed on Sf21 cells were expressed in the correct conformation and presented epitopes recognizable by these anti-TSA56 antibodies.

### Cell-Based ELISA Detected ST-Positive Antisera With High Accuracy

After confirming that recombinant antigens on Sf21 cells could interact with antibodies in ELISA, we examined the feasibility of using this system for ST diagnosis by assessing serum samples obtained from field-caught rats. We collected a total of 69 serum samples from rat species caught on the Penghu and Lanyu islands of Taiwan and confirmed ST antibody reaction in those samples by means of IFA **(** **Table 2**). To establish a cutoff for cell-based ELISA detection, 13 ST-negative sera consisting of 4 normal rat sera and 9 IFA-confirmed ST-negative field-caught rat sera were tested against all recombinant baculovirus-infected cells (i.e., infected with the four recombinant viruses, as well as wild-type virus) in ELISA. The mean value for the resulting ELISA reads plus two standard deviations (0.056) was set as the cutoff for our cell-based ELISA ST detection system.

**Figure 6:**
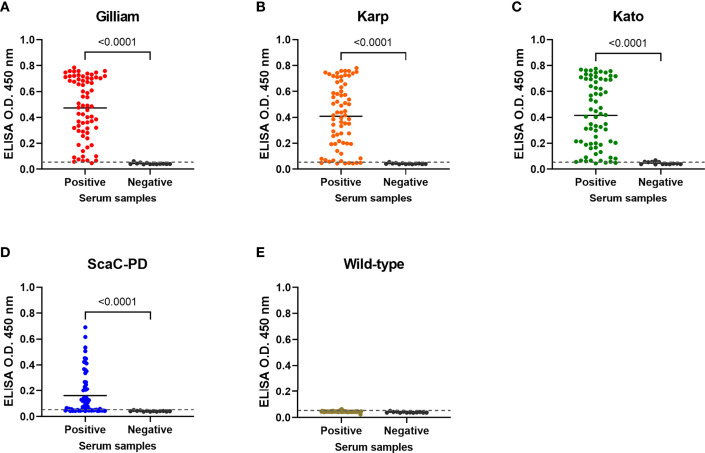
Cell-based ELISA detection of ST in serum samples obtained from field-caught or non-ST rats. Sixty-nine rat sera confirmed as having been infected with *O. tsutsugamushi* by IFA (Positive) and thirteen negative control rat sera (Negative) were subjected to cell-based ELISA using cells displaying Gilliam TSA56 **(A)**, Karp TSA56 **(B)**, Kato TSA56 **(C)**, and ScaC-PD **(D)** antigens, and cells infected with wild-type baculovirus **(E)**. Individual data points are shown and the solid line represents the mean value. Dotted line: cutoff value of 0.056 determined as the mean value of negative rat serum reactivities against each of the antigens plus two standard deviations. *P*-values determined by Welch’s *t*-test are displayed above the plots.

On average, the 69 positive sera presented significant ELISA signal relative to the 13 negative sera in cell-based ELISA using Gilliam, Karp, and Kato TSA56 and ScaC-PD antigens, though the four antigens exhibited differential discriminant capacities in detecting the 69 rat sera **(** **Figures 6A–D** **)**. In contrast, the 69 positive sera showed indistinguishable signals to negative sera in ELISA using cells infected by wild-type baculovirus (**Figure 6E**). Most IFA-positive serum samples reacted with the three TSA56 proteins, but reactivity with ScaC-PD was less pronounced **(** **Table 2** **)**. Only one “IFA-positive” serum sample presented a signal lower than the cutoff and, accordingly, was defined as ST-negative when reacted with TSA56 antigen from the Gilliam strain. Numbers of false-negative sera increased to 7, 4, and 28 when the sera were interacted with Karp, Kato, or ScaC-PD antigens, respectively. Among the 13 IFA-negative sera, 1 or 2 sera became false-positive in ELISA according to our cutoff value when using TSA56 antigen from Gilliam or Kato, respectively **(** **Tables 2** and **3** **)**.

**Table 2:** Diagnostic results for each rat serum sample upon reaction with individual antigens in the cell-based ELISA.

Sample No.	Host species	IFA	Gilliam	Karp	Kato	ScaC-PD
PH20-2	*Rattus norvegicus*	**+**	**+**	**+**	**+**	**−**
PH20-3	*Rattus norvegicus*	**+**	**+**	**+**	**+**	**−**
PH20-4	*Rattus norvegicus*	**++**	**+**	**+**	**+**	**+**
PH20-5	*Rattus norvegicus*	**++**	**+**	**+**	**+**	**+**
PH20-6	*Rattus losea*	**++**	**+**	**+**	**+**	**+**
PH20-7	*Rattus losea*	**++**	**+**	**+**	**+**	**−**
PH20-20	*Rattus losea*	**++**	**+**	**+**	**+**	**−**
PH20-21	*Rattus losea*	**++**	**+**	**+**	**+**	**+**
PH20-22	*Rattus losea*	**++**	**+**	**+**	**+**	**−**
PH20-23	*Rattus norvegicus*	**++**	**+**	**+**	**+**	**+**
PH20-32	*Rattus losea*	**++**	**+**	**+**	**+**	**+**
PH20-39	*Rattus losea*	**++**	**+**	**+**	**+**	**−**
PH20-40	*Rattus losea*	**+**	**+**	**+**	**+**	**−**
PH20-41	*Rattus losea*	**++**	**+**	**+**	**+**	**+**
PH20-44	*Rattus losea*	**++**	**+**	**+**	**+**	**+**
PH20-45	*Rattus losea*	**++**	**+**	**+**	**+**	**−**
PH20-46	*Rattus losea*	**++**	**+**	**+**	**+**	**+**
PH20-48	*Rattus losea*	**++**	**+**	**−**	**−**	**−**
PH20-49	*Rattus losea*	**+**	**+**	**+**	**+**	**+**
PH20-50	*Rattus losea*	**++**	**+**	**+**	**+**	**−**
PH20-51	*Rattus losea*	**++**	**+**	**−**	**−**	**−**
PH20-52	*Rattus losea*	**+**	**−**	**−**	**−**	**−**
PH20-53	*Rattus losea*	**+**	**+**	**+**	**+**	**−**
PH20-54	*Rattus losea*	**++**	**+**	**+**	**+**	**−**
PH20-55	*Rattus norvegicus*	**++**	**+**	**+**	**+**	**−**
PH20-56	*Rattus losea*	**+**	**+**	**+**	**+**	**+**
PH20-57	*Rattus losea*	**++**	**+**	**+**	**+**	**−**
PH20-62	*Rattus losea*	**+**	**+**	**+**	**+**	**+**
OI07-2	*Rattus mindanaoensis*	**++**	**+**	**+**	**+**	**+**
OI07-3	*Rattus mindanaoensis*	**++**	**+**	**+**	**+**	**+**
OI07-4	*Rattus mindanaoensis*	**++**	**+**	**+**	**+**	**+**
OI07-5	*Rattus mindanaoensis*	**++**	**+**	**+**	**+**	**+**
OI07-6	*Rattus mindanaoensis*	**++**	**+**	**+**	**+**	**+**
OI07-7	*Rattus mindanaoensis*	**++**	**+**	**+**	**+**	**+**
OI07-8	*Rattus mindanaoensis*	**++**	**+**	**+**	**+**	**+**
OI07-9	*Rattus mindanaoensis*	**++**	**+**	**+**	**+**	**−**
OI07-10	*Rattus mindanaoensis*	**++**	**+**	**+**	**+**	**+**
OI07-11	*Rattus mindanaoensis*	**++**	**+**	**+**	**+**	**+**
OI07-12	*Rattus mindanaoensis*	**++**	**+**	**+**	**+**	**+**
OI07-13	*Rattus mindanaoensis*	**++**	**+**	**+**	**+**	**+**
OI07-14	*Rattus mindanaoensis*	**++**	**+**	**+**	**+**	**+**
OI07-15	*Rattus mindanaoensis*	**++**	**+**	**+**	**+**	**−**
OI07-16	*Rattus mindanaoensis*	**++**	**+**	**+**	**+**	**+**
OI07-17	*Rattus mindanaoensis*	**++**	**+**	**+**	**+**	**+**
OI07-18	*Rattus mindanaoensis*	**++**	**+**	**+**	**+**	**+**
OI07-19	*Rattus mindanaoensis*	**++**	**+**	**+**	**+**	**−**
OI07-20	*Rattus mindanaoensis*	**++**	**+**	**+**	**+**	**+**
OI07-21	*Rattus mindanaoensis*	**++**	**+**	**+**	**+**	**+**
OI07-22	*Rattus mindanaoensis*	**++**	**+**	**+**	**+**	**+**
OI07-23	*Rattus mindanaoensis*	**++**	**+**	**+**	**+**	**−**
OI07-24	*Rattus mindanaoensis*	**++**	**+**	**−**	**+**	**−**
OI07-25	*Rattus mindanaoensis*	**++**	**+**	**+**	**+**	**−**
OI07-26	*Rattus mindanaoensis*	**++**	**+**	**+**	**+**	**+**
OI07-27	*Rattus mindanaoensis*	**++**	**+**	**+**	**+**	**+**
OI07-28	*Rattus mindanaoensis*	**++**	**+**	**+**	**+**	**−**
OI07-29	*Rattus mindanaoensis*	**++**	**+**	**−**	**+**	**+**
OI07-30	*Rattus mindanaoensis*	**++**	**+**	**−**	**+**	**+**
OI07-31	*Rattus mindanaoensis*	**++**	**+**	**+**	**+**	**−**
OI07-32	*Rattus mindanaoensis*	**++**	**+**	**+**	**+**	**+**
OI07-33	*Rattus mindanaoensis*	**++**	**+**	**+**	**+**	**+**
OI07-34	*Rattus mindanaoensis*	**++**	**+**	**+**	**+**	**+**
OI07-35	*Rattus mindanaoensis*	**++**	**+**	**−**	**+**	**−**
OI07-36	*Rattus mindanaoensis*	**++**	**+**	**+**	**+**	**−**
OI07-37	*Rattus mindanaoensis*	**++**	**+**	**+**	**+**	**+**
OI07-38	*Rattus mindanaoensis*	**++**	**+**	**+**	**+**	**+**
OI07-39	*Rattus mindanaoensis*	**++**	**+**	**+**	**+**	**+**
OI07-40	*Rattus mindanaoensis*	**++**	**+**	**+**	**+**	**−**
OI07-41	*Rattus mindanaoensis*	**++**	**+**	**+**	**+**	**+**
OI07-42	*Rattus mindanaoensis*	**++**	**+**	**+**	**−**	**−**
TN08-35	*Rattus norvegicus*	**−**	**−**	**−**	**−**	**−**
TN08-38	*Rattus norvegicus*	**−**	**−**	**−**	**−**	**−**
TN08-39	*Rattus norvegicus*	**−**	**−**	**−**	**−**	**−**
TN08-41	*Rattus norvegicus*	**−**	**−**	**−**	**−**	**−**
TN08-44	*Rattus norvegicus*	**−**	**−**	**−**	**−**	**−**
TN08-45	*Rattus norvegicus*	**−**	**−**	**−**	**−**	**−**
TN08-47	*Rattus norvegicus*	**−**	**−**	**−**	**+**	**−**
TN08-48	*Rattus norvegicus*	**−**	**−**	**−**	**−**	**−**
TC01-14	*Rattus losea*	**−**	**+**	**−**	**+**	**−**
Normal-1	Narl : LE (Long Evans)	**−**	**−**	**−**	**−**	**−**
Normal-2	Narl : LE (Long Evans)	**−**	**−**	**−**	**−**	**−**
Normal-3	Narl : LE (Long Evans)	**−**	**−**	**−**	**−**	**−**
Normal-4	Narl : LE (Long Evans)	**−**	**−**	**−**	**−**	**−**

**Table 3:** Detection efficiency of our cell-based ELISA system with individual or combined antigens for the 69 positive and 13 negative serum samples.

Parameters	Gilliam	Karp	Kato	ScaC-PD	Combined
True positive (TP)	68	62	65	41	68
True negative (TN)	12	13	11	13	11
False positive (FP)	1	0	2	0	2
False negative (FN)	1	7	4	28	1
Positive predict value	0.99	1.00	0.97	1.00	0.97
Negative predict value	0.92	0.65	0.73	0.32	0.92
Sensitivity (%)	98.6	89.9	94.2	59.4	98.6
Specificity (%)	92.3	100.0	84.6	100.0	84.6
Accuracy (%)	97.6	91.5	92.7	65.9	96.3

To ensure the specific reaction of ST-positive sera to recombinant antigens presenting on insect cells, we selected each of the six positive sera that were determined as positive when interacting with individual antigens, as well as five negative sera, and performed a titration assay (**Figure S1**). Serum antibody titers derived from the assay were strongly correlated with ELISA optical density (O.D.) values for 1:100-diluted sera (**Figure S2**), supporting that the dilution factor (1:100) we used was appropriate for detecting all serum samples. In terms of the efficiency of our cell-based ELISA with individual antigens, Gilliam and Kato TSA56 gave relative higher sensitivities of 98.6% and 94.2%, whereas Karp TSA56 and ScaC-PD presented high specificity (100%) **(** **Table 3**). Defining a sample as positive if it reacted with any of the four antigens, the sensitivity and specificity of our cell-based ELISA system (incorporating all four antigens) is 98.6% and 84.6%, respectively, and diagnostic accuracy is 96.3% **(** **Table 3**, column **“**Combined”**)**.

Our cell-based ELISA system presented superior diagnostic capacity for ST in rat sera, indicating that it can assist field surveys of ST-spreading natural hosts. To test if this system can be applied to ST diagnosis of human serum samples, we performed a pilot study with nine human serum samples. Five samples (18A001, 18A002, 18A004, 18A006, and 19A002) were defined as ST-negative by IFA, and the remaining four (18A003, 18A005, 19A004, and 19A008) were defined as ST-positive. We determined a cutoff value (0.280) as the mean plus two standard deviations of reads from the five negative sera tested against all recombinant baculovirus-infected cells (i.e., infected with the four recombinant viruses, as well as wild-type virus). Only the ST-negative sample 18A002 exceeded this cutoff for Gilliam antigen, thus representing a false-positive (**Table 4**). However, all four ST-positive sera were detected as true-positive by our cell-based ELISA using at least one of the TSA56 antigens (**Table 4**). Using IFA results as a reference, the sensitivity and specificity of our cell-based ELISA platform (combining all four antigens) for human sera is 100% and 80%, respectively. The diagnostic accuracy is 88.9% (**Table 5**).

**Table 4:** A pilot study using our cell-based ELISA system to diagnose human patients’ serum samples.

Sample No.	IFA	Gilliam	Karp	Kato	ScaC-PD
18A001	**−**	**−**	**−**	**−**	**−**
18A002	**−**	**+**	**−**	**−**	**−**
18A004	**−**	**−**	**−**	**−**	**−**
18A006	**−**	**−**	**−**	**−**	**−**
19A002	**−**	**−**	**−**	**−**	**−**
18A003	**+**	**+**	**−**	**+**	**+**
18A005	**+**	**+**	**−**	**−**	**−**
19A004	**+**	**−**	**−**	**+**	**−**
19A008	**+**	**+**	**+**	**−**	**−**

**Table 5:** Detection efficiency of the cell-based ELISA system in human serum sample diagnosis.

Parameters	Gilliam	Karp	Kato	ScaC-PD	Combined
True positive (TP)	3	1	2	0	4
True negative (TN)	4	5	5	5	4
False positive (FP)	1	0	0	0	1
False negative (FN)	1	3	2	4	0
Positive predict value	0.75	1.00	1.00	–	0.80
Negative predict value	0.80	0.63	0.71	0.56	1.00
Sensitivity (%)	75.0	25.0	50.0	0.0	100.0
Specificity (%)	80.0	100.0	100.0	100.0	80.0
Accuracy (%)	77.8	66.7	77.8	55.6	88.9

## Discussion

Currently, laboratory-based diagnosis of ST relies on culturing live bacteria in mouse cell lines and then assaying interaction of patient serum with those cell cultures by means of IFA, which is both biohazardous and time-consuming (8). As an alternative, purified antigens produced in heterologous systems can be used in low BSL laboratories. However, purifying, refolding, and coating such recombinant antigens on microtiter plates are labor-intensive processes. Cell-based assays using antigens expressed on cell surfaces can avoid these complex manufacturing processes. A cell-based IFA using baculovirus-infected insect cells expressing TSA56 has been developed for ST diagnosis and it showed high consistency in terms of serological results with conventional IFA (31). As an extension of that approach, we displayed *O. tsutsugamushi* antigens on insect cell surfaces and established a cell-based ELISA system. ST detection can be quantified *via* photocytometry or be evaluated by color development, thereby enhancing the convenience of ST serological diagnosis.

To properly express TSA56, we referred to previous studies in order to design the cloning fragments. TSA56 has two TM domains, so most studies remove at least one of them for heterologous expression of this protein. Previously, to act as antigen for ST serology tests, TSA56 has been cloned from amino acid residue 84/85 to the terminus (12, 16, 32), from residues 80 to 459 (11), or from residues 31 to 274 (14). Even a fragment solely comprising residues 411–423 has been deployed as antigen (33). Those studies demonstrate that the extracellular portion of TSA56 is most antigenic for serological diagnoses. It is worth noting that most of the aforementioned cloning strategies shared the same drawback in that the recombinant proteins were expressed in inclusion bodies, even for the shortest peptide (33), so protein denaturing and refolding steps were necessary for protein purification. Since the antigens did not need to be purified in our cell-based ELISA, we expressed full-length TSA56 to ensure display of the correct TSA56 conformation for serum antibody recognition.

In addition to TSA56, we included a ScaC antigen in our cell-based ELISA. In a previous study that used recombinant TSA56, 47-kDa protein, and several Sca proteins for ST ELISA, low background noise and significant ELISA signals for TSA56, ScaA, and ScaC were observed upon reaction with patient sera (16). However, ScaC represents the best universal antigen among them because it displays 77.4%−97.5% amino acid sequence identity among the Gilliam, Karp, Kato, and Boryong strains, whereas sequence identities of ScaA, ScaD, and ScaE only range from ~62.8 to 93.8% (16). Although ScaC is a transmembrane protein and its autotransporter domain facilitates folding of its passenger domain (15), expression of a recombinant construct lacking both TM and autotransporter domains did not affect ELISA outcome or immunogenicity in animal immunization experiments (34). We applied a similar strategy for ScaC of the Gilliam strain in the present study, but fused the TM-truncated passenger domain with GP64 6MC for better anchorage on Sf21 cell membranes, as demonstrated in previous studies (20, 21).

We found that all three TSA56 antigens and the SacC-PD antigen were properly expressed and displayed on insect cells upon recombinant virus infection **(** **Figures 3** and **4** **)**. Interestingly, TSA56 from the Gilliam strain exhibited relatively weak fluorescent signals in IFA **(** **Figure 4** **)** and weak absorbance signals in cell-based ELISA **(** **Figure 5B** **)** when detected by anti-His antibody. In contrast, this displayed antigen exhibited strong signals when detected by TSA56-specific antibodies **(** **Figure 5C** **)**, and presented the greatest sensitivity in detecting ST-positive rat sera relative to the other antigens **(** **Figure 6A**, **Tables 2** and **3** **)**. This outcome indicates that the His-tag may be inadequately exposed in the Gilliam TSA56 protein structure, so it is only partially recognized by anti-His antibody. Our cell-based ELISA did not generate additional background noise when used on normal (ST-negative) rat sera. The thirteen negative rat sera we tested generated a mean ELISA signal of 0.043 from reactions with all virus-infected cells, i.e., similar to the background values of 0.046~0.053 determined using purified protein as antigen (14). We calculated a cutoff value of 0.056 by adding two standard deviations to the mean value (10, 16, 29). Assessing additional serum samples from normal rats (or from field-caught rats determined to be ST-negative) may give a more credible cutoff value, but the one we have applied herein already displays good discriminant capacity.

According to a field investigation of small wild mammals with ST in 2011 in Taiwan, the Karp strain is the most widely distributed among rodents, followed by the TA763, Kato, and Gilliam strains (25). The Centers for Disease Control and Prevention of Taiwan have also reported that Karp is the most prevalent genotype in sera of ST patients (35). A worldwide serological analysis revealed that ~50% of ST isolates are serum-reactive to the Karp strain, and ~25% are serum-reactive to the Gilliam strain (2). In diagnostic systems using short peptides as antigen, TSA56 peptides from multiple strains had to be expressed to ensure detection of infection by different *O. tsutsugamushi* strains (33). In the present study, our ELISA results show that most antisera reacted with two to three of the TSA56 variant antigens **(** **Figures 6A–C**, **Tables 2** and **3** **)**, perhaps because we displayed full-length TSA56 on cell surfaces and the first 84 residues of TSA56 are highly conserved and significantly recognized by host immune systems (32, 36). Broad antigen cross-reactivity is a strong foundation for highly sensitive ST diagnostic assays (10). Our results indicate that full-length TSA56 may serve as an excellent universal antigen for diagnosing infections caused by different strains and we found that it performed even better than our theoretically universal antigen ScaC-PD.

Apart from rat sera, we also tested ST diagnosis against human serum samples (**Tables 4** and **5**). We consider this experiment a pilot study given the relatively small number of samples. Human sera exhibited higher background signals than rat sera, as evidenced by the higher cutoff value. Similar or higher cutoff values have been reported in other studies (9, 16, 37). We detected a false-positive when Gilliam TSA56 was used as antigen. Unlike our rat serum data that all true-positive rat sera interacted with the Gilliam TSA56 antigen, one human ST-positive sample (19A004) was solely recognized by the Kato TSA56 antigen (**Table 4**), indicating that multiple TSA56 antigens may be necessary for human serum testing. Nonetheless, we believe that expanding our pilot study to include more human ST samples may more accurately establish the detection efficiency of our platform for human diagnosis.

Baculovirus has long been used as an efficient large-scale protein expression system (19, 38, 39). Our study demonstrates that display of recombinant bacterial TSA56 and ScaC-PD on insect cell membrane *via* baculovirus infection can be used as a novel and convenient cell-based ELISA system for accurate, sensitive and specific ST detection. We are now testing this system on more sera from human ST patients. Owing to its advantages of biosafety, no requirement for specialized equipment, and easy operation, our cell-based ELISA system has the potential to become an efficient diagnostic platform for ST.

## Data Availability Statement

The original contributions presented in the study are included in the article/**Supplementary Material**. Further inquiries can be directed to the corresponding authors.

## Ethics Statement

The studies involving human participants were reviewed and approved by the Institutional Review Board of Tri-Service General Hospital. The patients/participants provided their written informed consent to participate in this study. The animal study was reviewed and approved by the Institutional Animal Care and Use Committee (IACUC) of the Institute of Preventive Medicine, National Defense Medical Center, Taiwan.

## Author Contributions

C-CLiao, C-HT, C-CLin, and Y-CC conceived and designed the research. C-CLiao, H-RL, P-RL, and C-CLin collected data and contributed samples. C-CLiao, H-RL, and P-RL performed the research. C-CLiao, C-HT, H-RL, P-RL, C-CLin, and Y-CC analyzed the data. C-CLiao, C-HT, and Y-CC wrote the manuscript. All authors contributed to the article and approved the submitted version.

## Funding

This research was funded by grants MOST 109-2927-I-001-511, MOST 109-2321-B-033-001, MOST 109-2327-B-016-004, MOST 110-2923-B-001-001, and MOST 110-2313-B-001-010 from the Ministry of Science and Technology of Taiwan, ROC; IPM108-G1-2 from the Institute of Preventive Medicine, National Defense Medical Center; VTA110-A-4-1 from VGH, TSGH, AS Joint Research Program; and grants under the 2020 NBRP Translational Research Project, Academia Sinica, Taiwan, ROC.

## Conflict of Interest

The authors declare that the research was conducted in the absence of any commercial or financial relationships that could be construed as a potential conflict of interest.

## Publisher’s Note

All claims expressed in this article are solely those of the authors and do not necessarily represent those of their affiliated organizations, or those of the publisher, the editors and the reviewers. Any product that may be evaluated in this article, or claim that may be made by its manufacturer, is not guaranteed or endorsed by the publisher.
