# Corrigendum: Development of a Scrub Typhus Diagnostic Platform Incorporating Cell-Surface Display Technology

**DOI:** 10.3389/fimmu.2021.803807

**Published:** 2021-11-16

**Authors:** Chih-Chi Liao, Chih-Hsuan Tsai, Huei-Ru Lo, Pey-Ru Lin, Chang-Chi Lin, Yu-Chan Chao

**Affiliations:** ^1^ Institute of Molecular Biology, Academia Sinica, Taipei, Taiwan; ^2^ Institute of Preventive Medicine, National Defense Medical Center, Taipei, Taiwan; ^3^ Department of Entomology, National Chung Hsing University, Taichung, Taiwan; ^4^ Department of Entomology, College of Bioresources and Agriculture, National Taiwan University, Taipei, Taiwan; ^5^ Department of Plant Pathology and Microbiology, College of Bioresources and Agriculture, National Taiwan University, Taipei, Taiwan

**Keywords:** baculovirus surface display, cell-based ELISA, *Orientia tsutsugamushi*, scrub typhus, serological diagnosis

In the original article, there was a mistake in **Figure 6** as published. The title of **Figure 6D** should be “ScaC-PD” instead of “Kato”. The corrected **Figure 6** appears below.

**Figure 6:**
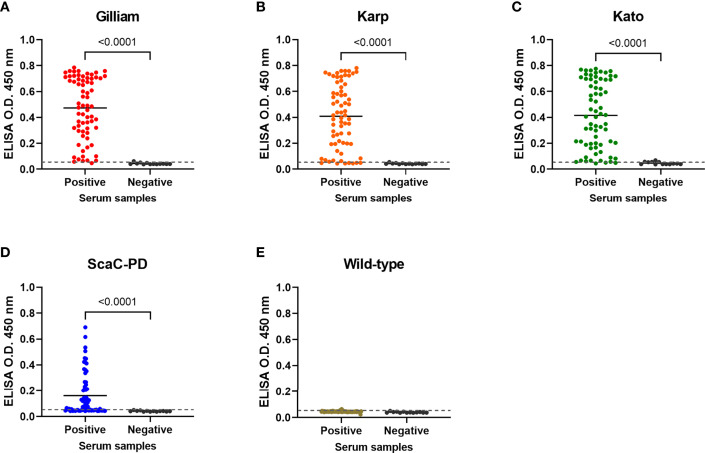
Cell-based ELISA detection of ST in serum samples obtained from field-caught or non-ST rats. Sixty-nine rat sera confirmed as having been infected with *O. tsutsugamushi* by IFA (Positive) and thirteen negative control rat sera (Negative) were subjected to cell-based ELISA using cells displaying Gilliam TSA56 **(A)**, Karp TSA56 **(B)**, Kato TSA56 **(C)**, and ScaC-PD **(D)** antigens, and cells infected with wild-type baculovirus **(E)**. Individual data points are shown and the solid line represents the mean value. Dotted line: cutoff value of 0.056 determined as the mean value of negative rat serum reactivities against each of the antigens plus two standard deviations. *P*-values determined by Welch’s *t*-test are displayed above the plots.

In the original article, there was an error in text. The term “His tag” should be “His-tag”.

A correction has been made to **Discussion**, paragraph 4:

“This outcome indicates that the His-tag may be inadequately exposed in the Gilliam TSA56 protein structure, so it is only partially recognized by anti-His antibody.”

The authors apologize for these errors and state that they do not change the scientific conclusions of the article in any way. The original article has been updated.

## Publisher’s Note

All claims expressed in this article are solely those of the authors and do not necessarily represent those of their affiliated organizations, or those of the publisher, the editors and the reviewers. Any product that may be evaluated in this article, or claim that may be made by its manufacturer, is not guaranteed or endorsed by the publisher.

