# Supplementary material for: Development of a Scrub Typhus Diagnostic Platform Incorporating Cell-Surface Display Technology
Source: Front Immunol. 2021 Oct 11;12:761136. doi: 10.3389/fimmu.2021.761136 (PMC8542878; doi:10.3389/fimmu.2021.761136)
Supplement: Supplementary file 1 [file DataSheet_1.docx]

Supplementary Material

## Supplementary Figures


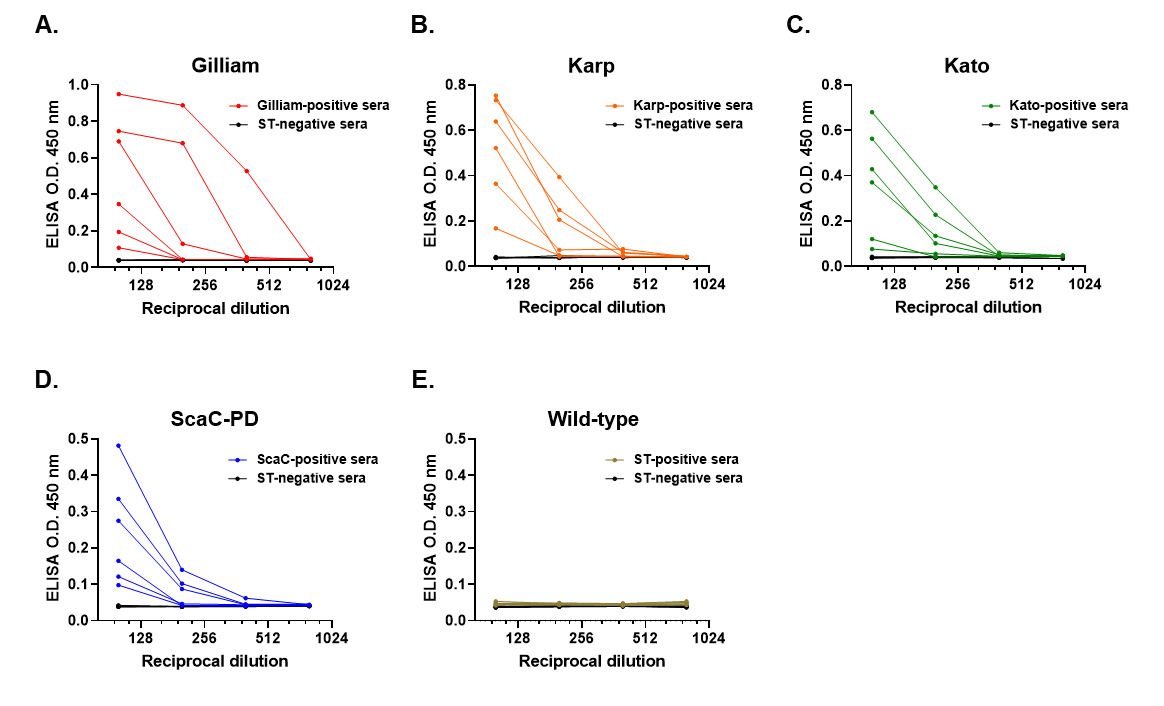


**Supplementary Figure 1. Determination of specific reactivities of ST sera to cell-based ELISA antigens.** Each of six rat sera that were positive to Gilliam TSA56 (A), Karp TSA56 (B), Kato TSA56 (C), and ScaC-PD (D) antigens and five ST-negative rat sera were two-fold serially diluted from 1:100 to 1:800 and applied to cell-based ELISA with the corresponding antigen. To validate background effects, six ST-positive sera were randomly selected from sera that were positive to any one of the recombinant antigens and applied to cell-based ELISA using Sf21 cells infected with wild-type baculovirus as antigens (five ST-negative rat sera were also included) (E). Individual lines represent ELISA signals from a serum sample.


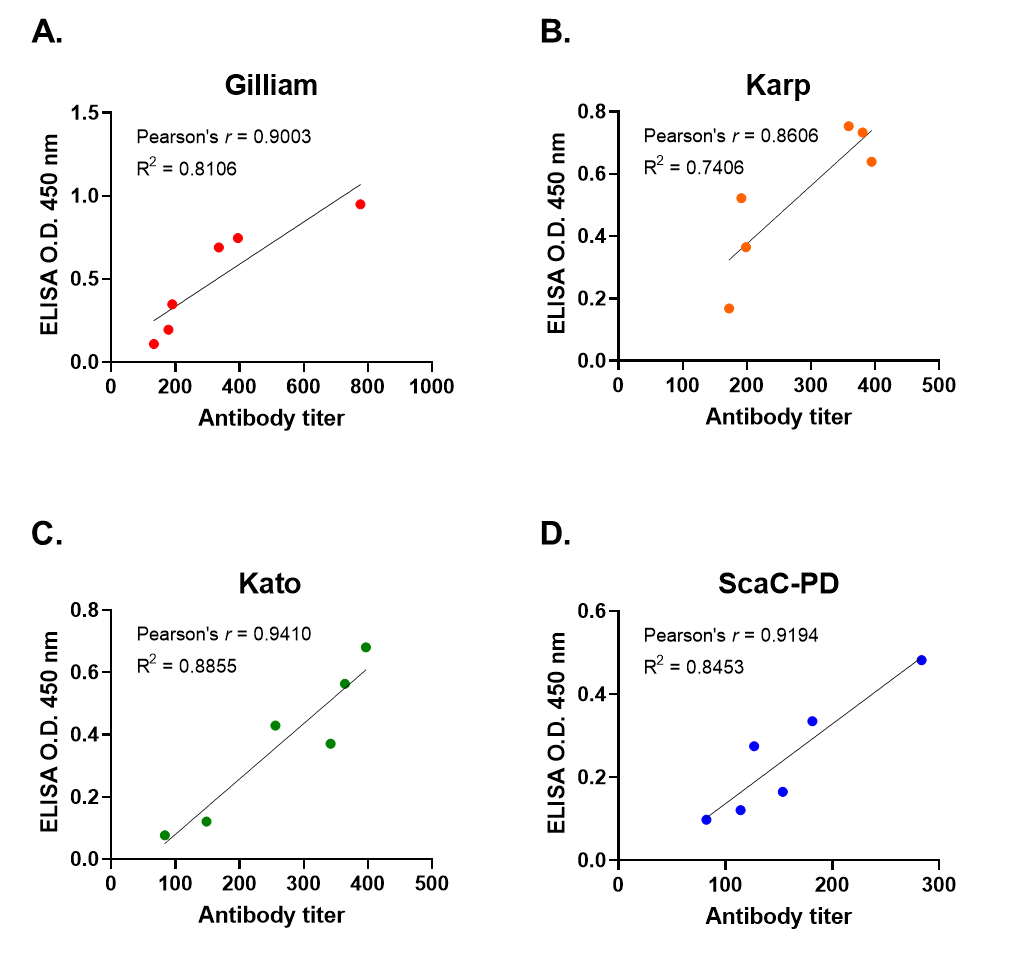


**Supplementary Figure 2.** **Correlation between ELISA O.D. values and antibody titers.** Antibody titer of the six rat sera that are positive of Gilliam TSA56 (A), Karp TSA56 (B), Kato TSA56 (C), and ScaC-PD (D), respectively, was determined by serial dilution and defined as the reciprocal of serum dilution giving twice the signal of the mean O.D. of negative sera. Linear regression and correlation analysis were performed using GraphPad Prism for ELISA signal from 1:100-diluted sera and their antibody titers. The Pearson correlation coefficient (Pearson’s *r*) and R^2^ value of linear regression were presented for each antigen system.
